# Efficacy of Corticosteroid Addition to a Periarticular Cocktail Injection to Counteract Nausea and Vomiting After Total Knee Arthroplasty

**DOI:** 10.7759/cureus.33874

**Published:** 2023-01-17

**Authors:** Toshihiro Ebihara, Takahiro Hamada, Kimitaka Nakamura, Akihiko Inokuchi, Teiyu Izumi, Ryuta Imamura, Takahiro Inoue, Hayato Inoue, Yosuke Kuroki, Takeshi Arizono

**Affiliations:** 1 Department of Orthopaedic Surgery, Kyushu Central Hospital of the Mutual Aid Association of Public School Teachers, Fukuoka, JPN

**Keywords:** triamcinolone acetonide, total knee arthroplasty, ponv, periarticular injection, corticosteroids

## Abstract

Background: Intraoperative periarticular injection of a "cocktail" of drugs is undertaken commonly in total knee arthroplasty (TKA). The addition of a corticosteroid to the periarticular injection is believed to offer greater pain relief because of its local anti-inflammatory effects, but the prevalence of postoperative nausea and vomiting (PONV) is not known. This retrospective observational study aimed to elucidate the relationship between corticosteroid addition to a periarticular cocktail injection (PCI) and PONV.

Materials and methods: Fifty-nine patients who underwent unilateral TKA for primary osteoarthritis were divided into two groups: corticosteroid and non-corticosteroid. The former had triamcinolone acetonide (40 mg) added to the same PCI. The primary outcome was the prevalence of nausea and vomiting within 48 hours following TKA.

Results: There was no significant difference between the two groups in terms of patient demographics. The overall prevalence of PONV was 16.9%. Fewer patients in the corticosteroid group complained of PONV than in the non-corticosteroid group (6.4% *vs*. 58.3%; p = 0.012).

Conclusions: The addition of a corticosteroid to a PCI suppressed PONV. Our results suggested that cocktail injections may have local and systemic effects.

## Introduction

Total knee arthroplasty (TKA) has been reported to be an efficacious and safe procedure for relieving pain and improving knee-joint function in patients with advanced osteoarthritis of the knee [1]. In general, a periarticular injection of a "cocktail" of drugs is administered around the joint capsule for postoperative analgesia [2]. Such periarticular cocktail injections (PCI) containing a corticosteroid are useful for postoperative analgesia and anti-inflammatory effects [3].

Many drugs used for cocktail injection have been reported [4]. However, side effects such as nausea are often a problem for strong analgesics such as morphine. Corticosteroids have been reported to be efficacious against nausea for patients being administered anticancer drugs [5], and for postoperative antiemetic effects if anticancer drugs are administered via the intravenous route [6]. On the other hand, there are few papers about the relationship between corticosteroid addition to a PCI and postoperative nausea and vomiting (PONV).

The purpose of this study was to examine the prevalence of PONV in a group given a corticosteroid and a group not administered a corticosteroid.

## Materials and methods

We retrospectively identified 59 (54 females and five males) patients who underwent unilateral TKA at our hospital from April 2018 to March 2020. The mean age was 77.9 (range, 60-90) years. The median body mass index (BMI) was 24.7 (range, 18.8-32.9) kg/m^2^.

All patients received the same spinal anesthetic and multimodal protocol to improve pain relief. Spinal anesthesia was administered by an anesthesiologist. Throughout the surgical procedure, anesthesia was maintained with propofol using a target-controlled device. We did not use regular antiemetics during the perioperative period.

We retrospectively identified 59 (54 females and five males) patients who underwent unilateral total knee arthroplasty (TKA) at our hospital from April 2018 to March 2020. The mean age was 77.9 (range, 60-90) years. The median BMI was 24.7 (range, 18.8-32.9) kg/m^2^.

All patients received the same spinal anesthetic and multimodal protocol to improve pain relief. Spinal anesthesia was administered by an anesthesiologist. Throughout the surgical procedure, anesthesia was maintained with propofol using a target-controlled device. We did not use regular antiemetics during the perioperative period.

All patients received a postoperative ultrasound-guided femoral nerve block (300 mL of physiologic (0.9%) saline solution containing ropivacaine (600 mg)). It was programmed to provide blockade at 5 mL/h, with an on-demand bolus infusion of 3 mL and a 30-minute lockout period.

The corticosteroid group comprised 47 patients who received a PCI with a corticosteroid. The non-corticosteroid group contained 12 patients who received a PCI without a corticosteroid because they had diabetes mellitus, rheumatoid arthritis, or previous bone and soft tissue infections. The PCI contained morphine hydrochloride (0.1 mg/kg), adrenaline (300 μg), ropivacaine hydrochloride hydrate (150 mg), and 40 mL of physiologic saline. The corticosteroid group received a PCI containing a corticosteroid (40 mg of triamcinolone acetonide). The non-corticosteroid group received a PCI that did not contain a corticosteroid.

Surgical procedures were carried out by a single senior surgeon. A standard medial parapatellar approach with a tourniquet was employed. The patella was resurfaced in all cases, and cement fixation was used for all components. Persona® (Zimmer Biomet, Warsaw, IN, USA) was implanted in all knees. Just before prosthesis implantation, the drug cocktail was injected into the posterior aspect of the capsule, the synovium, and around the collateral ligaments. The tourniquet was released to coagulate the bleeding point before prosthesis implantation. After capsule closure, all patients were administered an intra-articular injection of tranexamic acid (2 g) in physiologic saline (20 mL) to reduce blood loss. After wound closure, a knee bandage was applied. A drain was not inserted in any patient. The frequency of nausea and vomiting within 48 hours after surgery was confirmed by the medical record, and the frequency of occurrence was compared between the two groups.

Statistical analyses were undertaken using EZR (Easy R) 1.61 (www.jichi.ac.jp/saitama-sct/SaitamaHP.files/statmed.html). The Student’s t-test was used to compare the age, body weight, height, operative time, and sex, as well as the prevalence of postoperative fever and vomiting between the two groups.

This study was approved by the Kyushu Central Hospital Review Board on Clinical Research Plans (approval number 312) and conducted at Kyushu Central Hospital, Fukuoka, Japan.

## Results

The demographic characteristics between the two groups were not significantly different. Perioperative laboratory data (age, height, and BMI; intraoperative use of propofol; level of spinal anesthesia; systolic blood pressure; postoperative injection of flurbiprofen axetil; postoperative use of a diclofenac-sodium suppository; operative time) were not significantly different between the two groups. Risk factors for postoperative nausea and vomiting (PONV) did not affect nausea and vomiting (Table 1).

**Table 1:** Demographic data of the two groups Values are shown as the means (standard deviations). *Values are shown as the crude medians (interquartile range). PONV: postoperative nausea and vomiting; risk factors of PONV: pain, the medications used, anesthesia-related factors, and operative time [7].

	Steroid group	Non-steroid group	P-value
Age (years)	78.2(6.84)	76.9(5.68)	0.55
Height (cm)	152.6(7.28)	149.5(6.10)	0.18
Body mass index (kg/m^2^)	24.6(3.48)	25.3(3.77)	0.58
Intraoperative propofol*(ml)	262.5(176.5–360)	225(159.5–371)	0.86
Spinal anesthesia level (Th)	10.2(2.00)	9.8(3.60)	0.65
Intraoperative blood loss* (ml)	80(31–122.5)	76.5(49.75–134)	0.75
Systolic blood pressure (mmHg)	124.4(15.67)	129(13.43)	0.34
Postoperative flurbiprofen axetil injection* (times)	0(0–1)	1(0-1)	0.84
Postoperative diclofenac sodium suppository* (times)	0(0–2)	0(0-1)	0.17
Operative time (min)	101.5(14.11)	114.5(20.77)	0.01
Risk factors of PONV*	2(2–2)	2(2–2)	0.4

The prevalence of PONV was significantly higher in the non-corticosteroid group than in the corticosteroid group. The operative time was significantly different, whereas the use of propofol and the number of patients using non-steroidal anti-inflammatory drugs were similar between the two groups. We could collect 59 patients as a result. The overall prevalence of nausea and vomiting within 48 hours of TKA was 16.9%. The prevalence of nausea was three out of 47 cases in the corticosteroid group and seven out of 12 cases in the non-corticosteroid group. The prevalence of PONV was significantly lower in the corticosteroid group (p = 0.012) (Table 2). Univariate analysis showed significant differences in the prevalence of PONV, corticosteroid use, and operative time.

**Table 2:** Comparison of steroids group and non-steroids group PONV: postoperative nausea and vomiting

	Steroid group	Non-steroid group	Total amount	P value
PONV(+)	3	7	10	
PONV(−)	44	5	49	
Total amount	47	12	59	0.0012(<0.05)

A multivariate analysis was undertaken with PONV as the objective variable and corticosteroid use and operative time as explanatory variables. Only corticosteroid use had a significant antiemetic effect [OR (odds ratio) of 26.6, 95% CI (confidence intervals) 4.3-164.1, p<0.01]. On the other hand, operative time was not significantly different (OR 2.3, 95 % CI 0.4-14.3, p=0.36) (Table 3).

**Table 3:** Result of multivariate analysis PONV: postoperative nausea and vomiting; OR: odds ratio; CI: confidence intervals

	PONV (+)	Total cases (n)	OR (95%CI)	P-value
Steroids				
(+)	3	47	26.6(4.3–164.1)	<0.01
(−)	7	12		
Operative time (minute)				
≥102	5	30	2.3(0.4–14.3)	0.36
<101	5	29		

## Discussion

The factors that influence PONV are pain, the medications used, anesthesia-related factors, and operative time [8]. Apfel et al. suggested that the risk factors for PONV are being female, having a history of PONV/motion sickness, being a non-smoker, and postoperative use of opioids. The risk of PONV increased as the total number of risk factors increased. In their study, each additional risk factor increased the prevalence of PONV by ~20% up to 79% [7]. There was a significant difference in operative time between the two groups, but only corticosteroid use was significantly different in the multivariate analysis.

Several reports have focused on the analgesic effect of corticosteroids in a PCI. Some scholars reported an analgesic effect upon mixing a corticosteroid into a PCI, but other scholars did not find an analgesic effect. All reports evaluated pain using a visual analog scale [9-13]. We encourage the use of analgesics before intolerable pain arises. When we evaluated the frequency of analgesic use, there was no significant difference between the two groups.

We investigated whether a cocktail injection containing a corticosteroid had an antiemetic effect by recording the number of patients who complained of nausea or by using antiemetic agents. Yano et al. [14] and Ikeuchi et al. [11] reported that fewer patients complained of nausea in a group that received a cocktail injection containing a corticosteroid. Conversely, Deng et al. reported that corticosteroid-containing cocktail injections did not affect the prevalence of nausea and vomiting [15]. Moreover, they concluded that the addition of a corticosteroid to a cocktail injection would not increase the prevalence of postoperative infection, PONV, or any other complications. However, because the primary outcome was not PONV, risk factors were not adjusted in their study. We carried out multivariate analysis for the adjustment of risk factors, and the prevalence of PONV was significant in the corticosteroid group.

In general, corticosteroids are used during chemotherapy for the prevention of nausea or vomiting. However, the pharmacological mechanism of action of corticosteroids is still incompletely understood. The physiological effect of a corticosteroid is the result of its interaction with glucocorticoid receptors. Recent animal experiments have shown that glucocorticoid receptors on both sides of the nucleus of the solitary tract in the brainstem act to conduct the main antiemetic effect of a corticosteroid [16-18]. Several other pharmacological actions that require a corticosteroid to be antiemetic have been proposed, but experimental evidence is lacking [19]. A corticosteroid can reduce local inflammation effectively. A corticosteroid may reduce inflammation caused by afferent stimulation of the vomiting center in the dorsolateral medulla, thereby alleviating PONV [19]. In our study, the antiemetic effect of the corticosteroid was observed for 48 hours, even if administered locally. It has been suggested that corticosteroid-containing cocktail injections may have systemic effects upon local administration similar to those of intravenous administration, may diminish the side-effects of morphine (e.g., nausea), and, thus, have a long-acting effect.

The present study had three main limitations. First, this was a retrospective observational study, so the results revealed only an association between the corticosteroid mixed in a cocktail injection and PONV prophylaxis. Hence, we were not able to find a causal relationship. Second, although there was no difference in the risk factors for PONV between the two groups, the small number of patients in the non-corticosteroid group and the decision to use/not use a corticosteroid based on patient complications may have led to a selection bias. Third, the evaluation of pain was assessed by the frequency of analgesic use, which may have confounded the results. However, we encourage analgesic use if moderate pain is present, so we think that pain did not affect PONV.

## Conclusions

This retrospective observational study supported the addition of a corticosteroid to a cocktail injection because it reduced the risk of nausea and vomiting after TKA surgery and was beneficial for patients in the early postoperative period. The corticosteroid was efficacious for analgesia and against inflammation, but it also suppressed PONV. Our results suggest that cocktail injections may have local and systemic effects. RCTs with appropriate power to confirm the antiemetic efficacy of corticosteroids added to PAI are needed in the future.
